# SMITE: Single Molecule Imaging Toolbox Extraordinaire (MATLAB)

**DOI:** 10.21105/joss.05563

**Published:** 2023-10-02

**Authors:** David J. Schodt, Michael J. Wester, Mohamadreza Fazel, Sajjad Khan, Hanieh Mazloom-Farsibaf, Sandeep Pallikkuth, Marjolein B. M. Meddens, Farzin Farzam, Eric A. Burns, William K. Kanagy, Derek A. Rinaldi, Elton Jhamba, Sheng Liu, Peter K. Relich, Mark J. Olah, Stanly L. Steinberg, Keith A. Lidke

**Affiliations:** 1 Department of Physics and Astronomy, University of New Mexico, Albuquerque, United States of America; 2 Department of Mathematics and Statistics, Albuquerque, University of New Mexico, United States of America; 3 Department of Pathology, University of New Mexico Health Sciences Center, Albuquerque, United States of America

## Abstract

Fluorescence single molecule imaging comprises a variety of techniques that involve detecting individual fluorescent molecules. Many of these techniques involve localizing individual fluorescent molecules with precisions below the diffraction limit, which limits the spatial resolution of (visible) light-based microscopes. These methodologies are widely used to image biological structures at the nanometer scale by fluorescently tagging the structures of interest, elucidating details of the biological behavior observed.

Two common techniques are single-molecule localization microscopy (SMLM), (Betzig et al., 2006; Fazel & Wester, 2022; Hell, 2007; Lidke et al., 2005; Rust et al., 2006; van de Linde et al., 2011) which is used to produce 2D or 3D super-resolution images of static or nearly static structures, and single-particle tracking (SPT) (Shen et al., 2017), which follows the time course of one or a very small number of moving tagged molecules. SMLM often involves distributions of particles at medium to high density, while SPT works in a very low density domain. These procedures all require intensive numerical computation, and the methods are tightly interwoven.

## Statement of need

SMITE is a MATLAB-based toolbox that provides analysis tools for fluorescence single molecule imaging with an emphasis on single molecule localization microscopy (SMLM) and single-particle tracking (SPT). The SMITE toolbox consists of a MATLAB infrastructure with some C and CUDA code embedded to provide CPU/GPU speed-ups for particularly expensive computations. The source code for SMITE has been archived to GitHub: https://github.com/LidkeLab/smite

SMITE is designed around the concept that a parameter structure, the Single Molecule Fitting (SMF) structure, uniquely and completely defines the data analysis. The results are completely contained in a Single Molecule Data (SMD) structure. SMITE is designed to make lowest-level tools just as easy to use as the higher-level application-specific classes. All tools make use of the SMF and SMD structures. SMITE is organized into a set of namespaces that group similar tools and concepts. The namespace +smi contains the highest level tools that will be the most common entry point for processing SMLM and SPT data sets.

**Figure 1:**
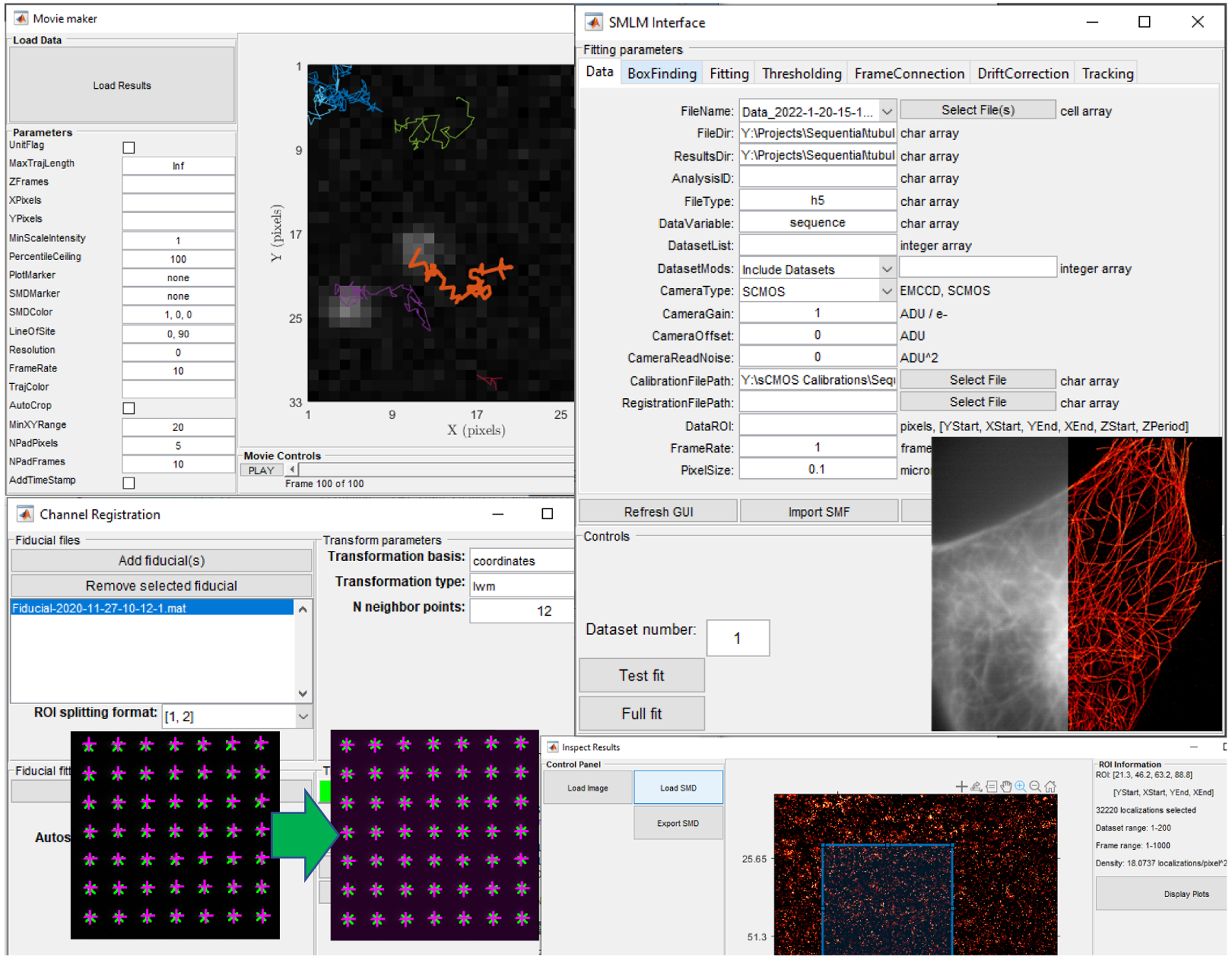
SMITE GUIs for (upper left) making movies from SPT trajectories, (upper right) SMLM analysis, (lower left) channel registration, and (lower right) inspection of results contained in SMD structures.

Code coverage includes mature SMLM data analysis techniques (applying gain and offset corrections to raw data, finding localizations, thresholding localizations based on various criteria, frame connection and drift correction), SMLM/SPT simulations, sophisticated SPT analyses, post-processing clustering and statistical analyses (e.g., diffusion analysis and hidden Markov models for characterizing dimers in SPT results), a variety of visualizations, experimental point spread function creation and characterization, all sprinkled with various examples of usage. Interaction with these tools is via GUIs or scripting. See Figure 1 for several examples of SMITE GUIs.

SMITE is a tool designed to be used by researchers and upper level students interested in fluorescence single molecule imaging and applications. Some of the algorithms have already been published: 2D Gaussian blob maximum likelihood estimate (Smith et al., 2010), frame connection (Schodt & Lidke, 2021), drift correction (Wester et al., 2021), Bayesian grouping of localizations (Fazel et al., 2022), diffusion estimation (Relich et al., 2016). However, this is the first time that they have been integrated together, sharing common data structures. Applications are described in (Bailey et al., 2022; Franco Nitta et al., 2021; Mazloom Farsibaf et al., 2021; Schodt et al., 2023). Typical raw image data can be found in (Pallikkuth, Martin, et al., 2018). A summary of the namespaces and classes in SMITE can be found in the online documentation at https://github.com/LidkeLab/smite/blob/main/doc/SMITEclasses.md.

SMAP (Ries, 2020), an alternative MATLAB integrated SMLM/SPT code, is GUI oriented, while SMITE was designed to be more focused on scripting (although many GUIs are available as well) in order to make batch processing extremely simple. SMITE, in addition, is designed to operate with HDF5 (Hierarchical Data Format) files which efficiently store very large datasets, while SMAP preferentially works with TIFF formatted files. Both SMITE and SMAP work with separate software to control instruments, MATLAB Instrument Control (MIC) (Pallikkuth, Meddens, et al., 2018) and Micro-Manager (Edelstein et al., 2014), respectively.
